# Oxidative Stability, Carcass Traits, and Muscle Fatty Acid and Amino Acid Profiles in Heat-Stressed Broiler Chickens

**DOI:** 10.3390/antiox10111725

**Published:** 2021-10-29

**Authors:** Mahmoud S. El-Tarabany, Omar A. Ahmed-Farid, Mohamed Abdo Nassan, Ayman S. Salah

**Affiliations:** 1 Department of Animal Wealth Development, Faculty of Veterinary Medicine, Zagazig University, Sharkia P.O. Box 44511, Egypt; 2 Physiology Department, National Organization for Drug Control and Research (NODCAR), Giza P.O. Box 35521, Egypt; ebntaimya@yahoo.com; 3 Department of Clinical Laboratory Sciences, Turabah University College, Taif University, P.O. Box 11099, Taif 21944, Saudi Arabia; m.nassan@tu.edu.sa; 4 Department of Animal Nutrition and Clinical Nutrition, Faculty of Veterinary Medicine, New Valley University, El-Kharga P.O. Box 72511, Egypt; asabry3999@yahoo.com

**Keywords:** broilers, heat stress, carcass, meat composition, oxidation

## Abstract

The objective was to elucidate the effects of chronic heat stress on carcass traits, muscle oxidative stability, muscle fatty acids and amino acid profiles in broiler chickens. A total of 100-day-old male Ross broiler chicks were divided into two equal groups of five replicates. The control group (TN) was maintained on a thermoneutral condition, while the experimental group (HS) was subjected to 8 h of heat stress (34 °C). The HS group showed lower dressing percentage and breast yield compared with the TN group (*p* = 0.040 and 0.042, respectively). Meanwhile, heat stress significantly increased the percentage of abdominal fat in broiler chickens (*p* = 0.001). The HS group showed significantly lower levels of PUFA (linoleic, docosahexaenoic and eicosapentaenoic) in the breast (*p* = 0.003, 0.002 and 0.001, respectively) and thigh (*p* = 0.001, 0.009 and 0.003, respectively) muscles than did the TN group. The levels of α-lenolinec acid in the breast and thigh muscles did not differ between both experimental groups (*p* = 0.818 and 0.060, respectively). With exception of threonine, tyrosine and phenylalanine, the levels of essential AA in the breast muscles were significantly (*p* ˂ 0.05) reduced in the HS group. The HS group showed significantly higher concentration of malondialdehyde (MDA) in the breast muscles (*p* = 0.032). Meanwhile, the concentration of MDA in the thigh muscles did not differ between both experimental groups (*p* = 0.149). Furthermore, the HS group showed significantly lower superoxide dismutase and catalase in heart tissues (*p* = 0.005 and 0.001, respectively). In conclusion, chronic thermal stress deteriorates carcass yield and the oxidative stability of breast muscles, as well as the levels of PUFA and essential AA in broiler chickens. However, the oxidative stability of thigh muscles was not affected.

## 1. Introduction

In the tropical and subtropical regions, heat stress is the main limiting factor of poultry industries [1]. It is also expected that global warming will increase the heat stress-related problems [2]. Unfortunately, the harmful effects of thermal stress to poultry health and production are likely to continue in the future. In this context, broilers are more liable to thermal stress due to their fast growth, rapid metabolic rate and high level of production [3]. As a compensatory mechanism to reduce the burden of heat stress, birds try to decrease the heat production by reducing feed intake and consequently reducing growth rate and profitability [4].

Heat stresses can be classified as acute or chronic depending on the duration and severity [5]. Previous studies suggested that acute thermal stress could reduce the quality attributes of breast muscles in broiler chickens [6]. However, the impacts of chronic thermal stress on the meat quality of broiler chickens are rarely discussed. Lu et al. [7] reported that heat-exposed Arbor Acres broilers had significantly reduced breast muscle proportion, suggesting that meat yield of broiler chickens was decreased by thermal stress. Others demonstrated an 11.99% decrease in the breast yield of Cobb broilers when exposed to 24 h of thermal stress [8]. Similarly, Shakeri et al. [9] observed a 16% reduction in the breast muscle yield when broilers were maintained under cyclic heat stress conditions. Lu et al. [10] also showed that chronic thermal stress changes the aerobic metabolism and the process of glycolysis and intramuscular fat deposition in broilers, resulting in poor meat quality and low consumer acceptability [11].

Lipid oxidation, a process during which meat lipids oxidize and interact with other meat constituents, causes deterioration in the quality of meat and causes undesirable effects on nutritive value [12]. Mujahid et al. [13] also indicated that acute thermal stress increases the oxidative damages to chicken skeletal muscles. In this context, heat stress accelerates the denaturation process of proteins and cell death and results in accumulation of reactive oxygen species (ROS) and sequential oxidative damages in tissues [14]. Hence, heat stress damages or disturbs mitochondria with subsequent changes in energy-metabolic pathway. It is now widely accepted that the metabolic changes in energy substance could alter the meat quality attributes in chickens [15]. Therefore, the objective of the current work was to investigate the impact of prolonged heat stress on carcass traits, muscle oxidative stability, muscle fatty acids and amino acid profiles in broiler chickens. 

## 2. Materials and Methods

### 2.1. Birds and Management

A total of 100 day-old male chicks (Ross strain) were divided into 2 equal groups of five replicates (10 birds/replicate). The brooding house was provided with fresh bedding materials (15 birds/m^2^) and birds had free access to feed and water. Digital heaters were used to provide the supplementary heat and to maintain a stable housing temperature (Diesel heater; Naganpuriya High Tech Farming Equipment, Indore, India). During the first week of age, both groups were brooded at 34 °C. Thereafter, temperature was gradually decreased to reach 23 °C at the day 21 of age. The first group (TN) was maintained on a thermoneutral condition (23 ± 1 °C), while the other group (HS) was subjected to 8 h of heat stress at 34 °C (08:00–16:00 and 23 ± 1 °C for the remaining time). The relative humidity was adjusted at 58 ± 3% and regular observation was carried out to monitor the stability of housing temperature and ventilation. A routine vaccination program against Newcastle disease and Gumboro disease was applied. In accordance with NRC [16], all birds were fed the mash starter (crude protein: 22.40%; ME: 12.3 MJ/kg) and grower-finisher diets (crude protein: 19.75%; ME: 12.9 MJ/kg) during the periods 1–21 days and 22–42 days of age, respectively. 

### 2.2. Carcass Traits

At the end of the experiment (42 days of age), three birds per replicate (15/group) were randomly selected, weighed (Sartorius 1202 MP balance with accuracy 0.01 g), and slaughtered. The slaughter technique was practiced according to the Islamic protocol (HALAL Slaughter) of the Malaysian institutes [17]. The main jugulars of birds were severed with sharp knives without using any anesthetics to achieve effective bleeding. After evisceration and removal of the internal organs, carcasses were allowed to effectively drain for 5 min and chilled at 2 °C for 30 min. The carcass yield (dressing percentage) was estimated as an actual carcass weight relative to the live body weight. Thereafter, carcasses were divided into different cuts (breast and legs). The relative weight of breast (m. Pectoralis major plus m. Pectoralis minor), legs, liver, heart and abdominal fat was calculated. 

### 2.3. Muscle Fatty Acid and Amino Acid Profiles

From the above chilled carcasses, the thigh and breast muscles were carefully dissected and any fat or connective tissues were removed. The lean muscle samples were directed to the chemical analyses of muscle fatty acids (FA) and amino acids (AA) profiles. A chloroform:methanol mixture (2:1) was used to extract the lipids from muscle samples and the free FA were purchased from Sigma–Aldrich (Sigma, St. Louis, MO, USA). The esterification of supernatant was performed by adding 2 mL of methanol:sulphuric acid mixture (95:5). The gas chromatography (Agilent Technologies—7890A GC) was used to finalize the analysis. The samples were injected into the GC set loop, using the SupelcoSP2330 column, (30 mm × 0.32 mm × 0.2 μm film thickness; Cat. No. 24073, Sigma–Aldrich, St. Louis, MO, USA). The conditions of flow rate through the GC column and the splitless injection mode were practiced according to the procedures described by Radwan and Ahmed [18]. The concentrations of muscle saturated FA, monounsaturated FA (MUFA) and polyunsaturated FA (PUFA) are expressed as g/100 g.

According to the modified procedures of Hughes et al. [19], the contents of free AA in the thigh and breast muscles were estimated. Each muscle sample (1 g) was mixed with 10 mL of trichloroacetic acid solution and homogenized for 1 min at 12,000 rpm. The homogenate was centrifuged for 10 min at 5000 rpm and then filtered through a fine membrane (0.45 μm thickness). The samples were re-dried by adding the drying mixture, which composed of triethylamine:methanol:1 M sodium acetate trihydrate (1:2:2). The derivatized samples and the standards of AA were injected into the HPLC Nova-Pak^TM^ C_18_ column (4 µm, 3.9 × 4.6 mm).

### 2.4. Determination of Muscle Malondialdehyde and Antioxidant Activity

In order to determine the malondialdehyde (MDA) concentrations, the breast and thigh muscles were homogenized in buffer solution and centrifuged at 700× *g* to collect a clear supernatant layer. The HPLC Agilent technology (HP 1100 series, Santa Clara, CA, USA) was used to quantify the concentration of AA in muscle samples [20]. The analytical column of Supelcosil C18 (5 µm particle and 80 A° pore size) was used at a flow rate of 1.5 mL/ min and 250 nm wavelength. Five heart samples were obtained from each group. Each homogenate heart sample was prepared in a 10 mM phosphate buffer (pH 7.4). Then, the suspension was centrifuged at 12,000× *g* for 10 min at 4 °C to collect the clear supernatant. The activities of superoxide dismutase (SOD) and catalase (CAT) in the supernatant were determined at two-minute intervals. 

### 2.5. Statistical Analysis

The data were analyzed by ANOVA procedures of the IBM SPSS software program (Version 16.0; IBM Corp., Armonk, NY, USA). The model included the fixed effects of the thermal treatment (two levels: TN and HS) and the random effect of experimental error. Body weight at day 21 of age was included as a covariate in the statistical model. The outputs are expressed as means and the standard error of means (SEM). 

## 3. Results

The effects of chronic heat stress on carcass traits of broiler chickens are illustrated in Table 1. The heat-stressed group showed lower carcass yield (dressing percentage) and breast yield compared with the thermoneutral group (*p* = 0.040 and 0.042, respectively). Meanwhile, heat stress increased the percentage of abdominal fat in broiler chickens (*p* = 0.001). The percentages of leg, liver and heart did not differ between both experimental groups (*p* = 0.104, 0.064 and 0.060, respectively).

The effects of chronic heat stress on the contents of FA in breast and thigh muscles of broiler chickens are described in Table 2 and Table 3. The prolonged thermal stress significantly increased the contents of saturated FA (myristic and palmitic) in breast (*p* = 0.001 and 0.012, respectively) and thigh (*p* = 0.018 and 0.006, respectively) muscles of broiler chickens. Meanwhile, chronic heat stress decreased the concentrations of MUFA (myristoleic, palmitoleic and oleic) in breast (*p* = 0.005, 0.012 and 0.007, respectively) and thigh (*p* = 0.003, 0.018 and 0.008, respectively) muscles of broiler chickens. Moreover, the HS group showed significantly lower levels of PUFA (linoleic, docosahexaenoic and eicosapentaenoic) in breast (*p* = 0.003, 0.002 and 0.001, respectively) and thigh (*p* = 0.001, 0.009 and 0.003, respectively) muscles than did the TN group. The concentrations of α-linolenic acid in the breast and thigh muscles did not differ between both experimental groups (*p* = 0.818 and 0.060, respectively). 

The effects of chronic heat stress on the concentrations of AA in breast and thigh muscles of broilers are illustrated in Table 4 and Table 5. Except for threonine, tyrosine and phenylalanine, the levels of other essential AA (lysine, leucine, isoleucine, valine and methionine) in the breast muscles were significantly (*p* ˂ 0.05) reduced in the HS group. Moreover, chronic thermal stress decreased the levels of all essential AA in the thigh muscles of broiler chickens (*p* ˂ 0.05). 

**Figure 1:**
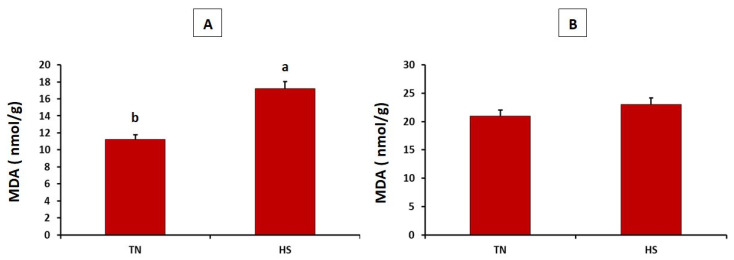
Effects of chronic heat stress on malondialdehyde (MDA) levels in breast (**A**) and thigh (**B**) muscles of broiler chickens (*p* = 0.032 and 0.149, respectively). ^a,b^ Values with different superscripts differ significantly.

**Figure 2:**
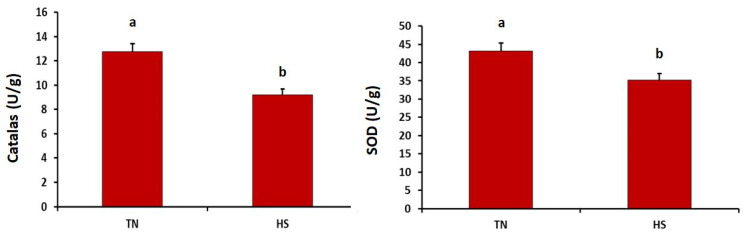
Effects of chronic heat stress on the activities of catalase and superoxide dismutase (SOD) in heart tissues of broiler chickens (*p* = 0.005 and 0.001, respectively). ^a,b^ Values with different superscripts differ significantly.

As described in Figure 1, the HS group showed significantly higher concentration of malondialdehyde in the breast muscles than did the thermoneutral group (*p* = 0.032). Meanwhile, the concentration of malondialdehyde in the thigh muscles did not differ between both experimental groups (*p* = 0.149). Furthermore, the HS group showed significantly lower superoxide dismutase and catalase in heart tissues (*p* = 0.005 and 0.001, respectively) (see Figure 2).

## 4. Discussion

It is believed that broiler chickens are more susceptible to adverse environmental conditions, probably due to continuous selection for the rapid growth rate [21]. However, the majority of the literature has focused on acute form or short duration thermal stress. Herein, the chronic thermal stress decreased the dressing percentage and breast yield in broiler chickens. Consistent with these findings, Mello et al. [8] demonstrated an 11.99% decrease in the breast yield of Cobb broilers when exposed to 24 h of thermal stress. They noticed that the legs yield was not influenced (*p* > 0.05) by the duration of thermal stress. Oliveira et al. [22] also stated that prolonged thermal stress decreased the breast yield by 9.5% in broilers. In this context, previous studies suggested that the reduction of breast yield may be attributed to the insufficient intake of nutrients and energy molecules, with a subsequent reduction in the synthesis and storage of glycogen in the breast muscles [23]. Others have assumed that the lower developmental rate of breast muscles may be related to the increased respiratory rate in heat-stressed broilers [24]. As a consequence, the activity of breast muscles is increased and glycogen reserves have been depleted.

It is believed that excessive abdominal fat is one of the major problems in the broiler industry [25]. Herein, chronic thermal stress increased the percentage of abdominal fat in Ross broilers. The increased deposition of abdominal fat in the HS group is probably an adaptive mechanism under hot environmental conditions, where dietary energy was stored as fat, and consequently the metabolic heat production is reduced. Consistent with these findings, some researchers observed that chronic heat exposure enhanced fat deposition in broiler chickens [26,27]. Lu et al. [7] also reported that prolonged exposure to thermal stress (34 °C) significantly increases the proportion of abdominal fat in BJY chickens. On the contrary, others reported a significant decrease in fat deposition due to thermal stress [28]. The above conflict may be attributed to age differences and breed of chickens, the model of thermal stress (constant or cyclic) and the duration of stressful conditions (acute or chronic).

The primary concern of thermal stress in the broiler industry is the adverse effect on meat composition and quality that influence consumer acceptability [29]. Moreover, heat stress increases the level of ROS and the related oxidative damages. Indeed, malondialdehyde (MDA) is a compound mainly produced when ROS attacks unsaturated lipid in muscles [30]. Unsurprisingly, prolonged thermal stress in the current study increased the concentration of MDA in the breast muscles of broiler chickens. This may indicate a shorter shelf life of breast muscle and the related manufactured products due to chronic thermal stress. However, the majority of reports have focused on the acute model of heat stress in broilers. Mujahid et al. [31] recorded more than two-fold increase of muscle MDA when broilers were exposed to acute heat stress. They suggested that high MDA level in the skeletal muscles may be due to the disturbed membrane function of mitochondria. Wang et al. [30] also stated that short duration of thermal stress (3 and 5 h) significantly increased the concentration of MDA in the pectoralis major of broiler chickens. After 7 days of thermal stress, the concentration of MDA in breast muscles of broilers was significantly increased [10]. Meanwhile, the differences in the levels of breast MDA disappeared when heat stress continued for 14 days. Interestingly, the chronic heat stress model in the current study did not affect the level of MDA in thigh muscles. The eminent oxidative stability of thigh muscle under chronic thermal stress may be due to an adaptive internal mechanism controlled by mitochondria to regulate ROS generation [32]. In this context, Hosseindoust et al. [33] suggested that the redness of leg muscles is usually associated with lower level of lipid peroxidation and reduced MDA activity.

Recently, there has been an increase in demand for healthy food products, including both the quantity and composition of lipids in broiler meat [34]. The present study demonstrated that prolonged heat stress increased the levels of saturated FA, but reduced the levels of MUFA and PUFA in breast and thigh muscles of Ross broilers. This may be attributed to the greater susceptibility of unsaturated FA to oxidative damage during the exposure to thermal stress [35]. Indeed, heat stress enhances the production of free radicals and ROS, with subsequent oxidative damages of lipid molecules [33] and modulation of lipogenic enzyme expression [36]. Herein, the higher contents of palmitic acid in breast and thigh muscles suggest a higher lipogenic activity in heat-stressed broilers, and this is usually associated with increased fat deposits in the abdomen [37]. Although PUFA tends to deteriorate upon initiation of oxidative stress, the levels of α-linolenic acid in breast and thigh muscles remains stable in heat-stressed broilers. In this context, Zhao et al. [38] tested whether thermal stress affects FA utilization in the muscles and measured the FA oxidation rate in the longissimus dorsi muscle of pigs. They reported a similar FA oxidation rate in both heat-stressed and thermoneueral groups. 

Heat stress is usually associated with accumulation of ROS in the mitochondria, with subsequent oxidative damages of proteins and DNA structures. When thermal stress is prolonged, the mitochondrial homeostasis is disturbed and ATP synthesis is decreased [39]. Except for threonine, tyrosine and phenylalanine, the present study noticed that chronic heat stress deteriorates the concentrations of essential AA in breast and thigh muscles of broiler chickens. This may be attributed to the increased corticosterone level in heat-stressed birds, which suppress protein synthesis and accelerate protein breakdown [40]. Furthermore, heat stress accelerates the depletion of amino acids as metabolic fuel to supply energy by liver gluconeogenesis [41]. Herein, the reduced mass of breast muscles in heat-stressed broilers may support this concept. Consistent with these findings, Pedroso et al. [42] noticed that prolonged stress increased catabolism of muscles to AA for supplying energy. Ma et al. [41] also demonstrated that thermal stress reduced the levels of essential AA (lysine, threonine, and glycine), suggesting a disturbance of muscle protein synthesis in heat-stressed broilers. Previous studies suggested that a deficiency of methionine increases fat deposition in broiler chickens [43]. Moreover, it has been observed that heat stress increases the methionine requirement of broilers [44]. In the current study, the reduced levels of muscle methionine in the HS group may explain the excessive deposition of abdominal fat.

Heat stress enhances the lipid peroxidation process and accumulation of ROS, and consequently exhausts the antioxidant defense system in broiler chickens [45]. Moreover, it is believed that both catalase and SOD act as the first line of the antioxidant defense system in the body tissues. In the current study, thermal stress reduced the levels of SOD and catalase in the heart tissues of broiler chickens. Consistent with these findings, Xue et al. [46] recorded a great depletion in serum antioxidant activity when Arbor Acres broiler chickens were exposed to cyclic thermal stress. Furthermore, others reported that short-term heat stress significantly decreased the activities of SOD and catalase in liver tissues of Pekin ducks [47].

## 5. Conclusions

It could be concluded that prolonged heat stress reduced the dressing percentage and breast yield, but increased the percentage of abdominal fat in broiler chickens. Moreover, chronic heat stress decreased the levels of MUFA (myristoleic, palmitoleic and oleic), PUFA (linoleic, docosahexaenoic and eicosapentaenoic), and essential AA in breast and thigh muscles of broilers. The oxidative stability of breast muscles was deteriorated in heat-stressed broiler chickens. Meanwhile, the oxidative stability of thigh muscles was not affected. The present results may be helpful to adjust the appropriate strategies to relieve burden of prolonged thermal stress in broiler chickens.

## Figures and Tables

**Table 1:** Effect of chronic heat stress on carcass traits of broiler chickens.

Parameters (%)	Group			
	TN ^1^	HS ^2^	SEM ^3^	*p*-Value
Dressing percentage	75.39	71.42	1.14	0.040
Breast	41.40	38.56	0.96	0.042
Legs	34.24	31.71	0.71	0.104
Liver	2.32	2.14	0.04	0.064
Heart	0.42	0.36	0.03	0.060
Abdominal fat	0.67	1.17	0.08	0.001

^1^ thermoneutral group; ^2^ thermal-stress group; ^3^ standard error of means.

**Table 2:** Effect of chronic heat stress on fatty acid profile (g/100 g) of breast muscles in broiler chickens.

Parameters	Group			
	TN ^1^	HS ^2^	SEM ^3^	*p*-Value
Myristic (C_14:0_)	0.79	0.95	0.03	0.001
Palmitic (C_16:0_)	31.07	36.31	0.95	0.012
Stearic (C_18:0_)	12.54	13.20	0.22	0.145
Myristoleic acid (C_14:1_)	1.12	0.91	0.04	0.005
Palmitoleic (C_16:1_)	1.21	0.94	0.05	0.012
Oleic (C_18:1_)	22.02	17.68	0.86	0.007
Linoleic (C_18:2n6_)	15.68	12.56	0.78	0.003
α-linolenic acid (C_18:3n3_)	0.91	0.92	0.03	0.818
Docosahexaenoic acid (C_22:6n3_)	0.60	0.45	0.02	0.002
Eicosapentaenoic acid (C_20:5n3_)	0.71	0.53	0.02	0.001

^1^ thermoneutral group; ^2^ thermal-stress group; ^3^ standard error of means.

**Table 3:** Effect of chronic heat stress on fatty acid profile (g/100 g) of thigh muscles in broiler chickens.

Parameters	Group			
	TN ^1^	HS ^2^	SEM ^3^	*p*-Value
Myristic (C_14:0_)	1.25	1.34	0.03	0.018
Palmitic (C_16:0_)	29.27	32.88	0.92	0.006
Stearic (C_18:0_)	11.05	12.22	0.35	0.224
Myristoleic acid (C_14:1_)	1.12	0.79	0.05	0.003
Palmitoleic (C_16:1_)	2.01	1.61	0.07	0.018
Oleic (C_18:1_)	29.46	20.98	1.32	0.008
Linoleic (C_18:2n6_)	20.45	13.69	0.47	0.001
α-linolenic acid (C_18:3n3_)	0.57	0.66	0.02	0.060
Docosahexaenoic acid (C_22:6n3_)	0.51	0.36	0.01	0.009
Eicosapentaenoic acid (C _20:5n3_)	0.84	0.58	0.04	0.003

^1^ thermoneutral group; ^2^ thermal-stress group; ^3^ standard error of means.

**Table 4:** Effect of chronic heat stress on amino acid profile (g/100 g) of breast muscles in broiler chickens.

Parameters	Group			
	TN ^1^	HS ^2^	SEM ^3^	*p*-Value
Lysine	7.52	5.88	0.36	0.017
Leucine	7.69	5.83	0.43	0.018
Isoleucine	3.26	2.54	0.17	0.012
Valine	3.95	3.18	0.18	0.016
Methionine	1.54	1.22	0.03	0.035
Tyrosine	2.30	1.83	0.11	0.083
Threonine	3.50	2.84	0.10	0.052
Phenylalanine	1.85	1.62	0.08	0.214
Histidine	2.69	2.23	0.13	0.028
Glycine	4.98	3.81	0.26	0.009
Proline	1.47	1.14	0.08	0.036
Arginine	4.99	3.86	0.18	0.057
Serine	2.61	2.14	0.12	0.055
Aspartic acid	8.55	6.71	0.44	0.022
Glutamic acid	11.29	9.40	0.65	0.157
Alanine	5.26	4.13	0.30	0.074

^1^ thermoneutral group; ^2^ thermal-stress group; ^3^ standard error of means.

**Table 5:** Effect of chronic heat stress on amino acid profile (g/100 g) of thigh muscles in broiler chickens.

Parameters	Group			
	TN ^1^	HS ^2^	SEM ^3^	*p*-Value
Lysine	7.32	5.87	0.37	0.024
Leucine	7.43	5.12	0.58	0.018
Isoleucine	3.23	2.29	0.21	0.001
Valine	4.28	2.93	0.15	0.008
Methionine	1.56	1.23	0.04	0.002
Tyrosine	2.25	1.77	0.11	0.001
Threonine	3.58	2.57	0.08	0.018
Phenylalanine	1.89	1.45	0.05	0.002
Histidine	2.78	2.09	0.12	0.006
Glycine	5.08	3.68	0.26	0.009
Proline	1.53	1.07	0.10	0.011
Arginine	4.91	3.62	0.16	0.015
Serine	2.83	1.88	0.05	0.001
Aspartic acid	7.78	3.36	0.21	0.003
Glutamic acid	11.72	8.57	0.41	0.009
Alanine	5.51	3.89	0.25	0.005

^1^ thermoneutral group; ^2^ thermal-stress group; ^3^ standard error of means.

## Data Availability

All data generated or analyzed during this study are included in this published paper.

## References

[1] Lin H., Jiao H.C., Buyse J., Decuypere E. (2006). Strategies for preventing heat stress in poultry. World’s Poult. Sci. J..

[2] Hansen J., Ruedy R., Sato M., Lo K. (2010). Global surface temperature change. Rev. Geophys..

[3] Zhang Z.Y., Jia G.Q., Zuo J.J., Zhang Y., Lei J., Ren L., Feng D.Y. (2012). Effects of constant and cyclic heat stress on muscle metabolism and meat quality of broiler breast fillet and thigh meat. Poult. Sci..

[4] Quinteiro-Filho W.M., Ribeiro A., Ferraz-de-Paula V., Pinheiro M.L., Sakai M., Sá L.R.M., Ferreira A.J.P., Palermo-Neto J. (2010). Heat stress impairs performance parameters, induces intestinal injury, and decreases macrophage activity in broiler chickens. Poult. Sci..

[5] Wang R.H., Liang R.R., Lin H., Zhu L.X., Zhang Y.M., Mao Y.W., Dong P.C., Niu L.B., Zhang M.H., Luo X. (2017). Effect of acute heat stress and slaughter processing on poultry meat quality and postmortem carbohydrate metabolism. Poult. Sci..

[6] Northcutt J.K., Foegeding E.A., Edens F.W. (1994). Water-holding properties of thermally preconditioned chicken breast and leg meat. Poult. Sci..

[7] Lu Q., Wen J., Zhang H. (2007). Effect of chronic heat exposure on fat deposition and meat quality in two genetic types of chicken. Poult. Sci..

[8] Mello J.L.M., Boiago M.M., Giampietro-Ganeco A., Berton M.P., Vieira L.D.C., Souza R.A., Ferrari F., Borba H. (2015). Periods of heat stress during the growing affects negatively the performance and carcass yield of broilers. Arch. Zootec..

[9] Shakeri M., Cottrell J., Wilkinson S., Ringuet M., Furness J., Dunshea F. (2018). Betaine and Antioxidants Improve Growth Performance, Breast Muscle Development and Ameliorate Thermoregulatory Responses to Cyclic Heat Exposure in Broiler Chickens. Animals.

[10] Lu Z., He X., Ma B., Zhang L., Li J., Jiang Y., Zhou G., Gao F. (2017). Chronic heat stress Impairs the quality of breast-muscle meat in broilers by affecting redox status and energy-substance metabolism. J. Agric. Food Chem..

[11] Lara L.J., Rostagno M.H. (2013). Impact of heat stress on poultry production. Animals.

[12] Shakeri M., Cottrell J.J., Wilkinson S., Le H.H., Suleria H.A., Warner R.D., Dunshea F.R. (2019). Growth performance and characterization of meat quality of broiler chickens supplemented with betaine and antioxidants under cyclic heat stress. Antioxidants.

[13] Mujahid A., Pumford N.R., Bottje W., Nakagawa K., Miyazawa T., Akiba Y., Toyomizu M. (2007). Mitochondrial oxidative damage in chicken skeletal muscle induced by acute heat stress. J. Poult. Sci..

[14] Fouad A.M., Chen W., Ruan D., Wang S., Xia W.G., Zheng C.T. (2016). Impact of heat stress on meat, egg quality, immunity and fertility in poultry and nutritional factors that overcome these effects: A review. Int. J. Poult. Sci..

[15] Yang Y., Wen J., Fang G.Y., Li Z.R., Dong Z.Y., Liu J. (2015). The effects of raising system on the lipid metabolism and meat quality traits of slow-growing chickens. J. Appl. Anim. Res..

[16] NRC (1994). Nutrient Requirements of Poultry.

[17] JAKIM (Department of Islamic Development Malaysia) (2011). Malaysian Protocol for the Halal Meat and Poultry Productions.

[18] Radwan O.K., Ahmed R.F. (2016). Amendment effect of resveratrol on diclofenac idiosyncratic toxicity: Augmentation of the anti-inflammatory effect by assessment of Arachidonic acid and IL-1β levels. J. Appl. Pharm. Sci..

[19] Hughes M.C., Kerry J.P., Arendt E.K., Kenneally P.M., McSweeney P.L.H., O’Neill E.E. (2002). Characterization of proteolysis during the ripening of semi-dry fermented sausages. Meat Sci..

[20] Karalas F., Karatepe M., Baysar A. (2002). Determination of free malondialdehyde in human serum by high performance liquid chromatography. Anal. Biochem..

[21] Geraert P.A., Guillaumin S., Leclercq B. (1993). Are genetically lean broilers more resistant to hot climate?. Br. Poult. Sci..

[22] Oliveira G.A., Oliveira R.F.M., Donzele J.L., Cecon P.R., Vaz R.G.M.V., Orlando U.A.D. (2006). Effect of environmental temperature on performance and carcass characteristics of broilers from 22 to 42 days old. Rev. Bras. Zootec..

[23] Rosa P.S., Faria Filho D.E., Dahlke F., Vieira B.S., Macari M., Furlan R.L. (2007). Performance and carcass characteristics of broiler chickens with different growth potential and submitted to heat stress. Braz. J. Poult. Sci..

[24] Faria Filho D.E., Rosa P.S., Figueiredo D.F., Dahlke F., Macari M., Furlan R.L. (2006). Low-protein diets on broilers performance reared under different temperatures. Pesqui. Agropecu. Bras..

[25] Bai S., Wang G., Zhang W., Zhang S., Rice B.B., Cline M.A., Gilbert E.R. (2015). Broiler chicken adipose tissue dynamics during the first two weeks post-hatch. Comp. Biochem. Physiol. A.

[26] Ain Baziz H., Geraert P.A., Guillaumin S. (1996). Chronic heat exposure enhances fat deposition and modifies muscle and fat partition in broiler carcasses. Poult. Sci..

[27] Geraert P.A., Padilha J.C.F., Guillaumin S. (1996). Metabolic and endocrine changes induced by chronic heat exposure in broiler chickens: Growth performance, body composition and energy retention. Br. J. Nutr..

[28] Smith M.O., Teeter R.G. (1993). Effects of feed intake and environmental temperature on chick growth and development. J. Agric. Sci..

[29] Zaboli G., Huang X., Feng X., Ahn D.U. (2019). How can heat stress affect chicken meat quality?—A review. Poult. Sci..

[30] Wang R.R., Pan X.J., Peng Z.Q. (2009). Effects of heat exposure on muscle oxidation and protein functionalities of pectoralis majors in broilers. Poult. Sci..

[31] Mujahid A., Akiba Y., Toyomizu M. (2009). Olive oil-supplemented diet alleviates acute heat stress-induced mitochondrial ROS production in chicken skeletal muscle. Am. J. Physiol. Regul. Integr. Comp. Physiol..

[32] Pamplona R., Costantini D. (2011). Molecular and structural antioxidant defenses against oxidative stress in animals. Am. J. Physiol. Regul. Integr. Comp. Physiol..

[33] Hosseindoust A., Oh S.M., Ko H.S., Jeon S.M., Ha S.H., Jang A., Son J.S., Kim G.Y., Kang H.K., Kim J.S. (2020). Muscle Antioxidant Activity and Meat Quality Are Altered by Supplementation of Astaxanthin in Broilers Exposed to High Temperature. Antioxidants.

[34] Gallardo M.A., Pérez D.D., Leighton F.M. (2012). Modification of fatty acid composition in broiler chickens fed canola oil. Biol. Res..

[35] Zulkifli I., Norma M.T.C., Israf D.A., Omar A.R. (2002). The effect of early-age food restriction on heat shock protein 70 response in heat-stressed female broiler chickens. Br. Poult. Sci..

[36] Missoten J., De Smet S., Raes K., Doran O. (2009). Effect of supplementation of the maternal diet with fi shoil or linseed oil on fatty acid composition and expression of D5- and D6- desaturase in tissues of piglets. Animal.

[37] Watkins B.A. (1991). Importance of essential fatty acids and their derivatives in poultry. J. Nutr..

[38] Zhao L., McMillan R.P., Xie G., Giridhar S.G., Baumgard L.H., El-Kadi S., Selsby J., Ross J., Gabler N., Hulver M.W. (2018). Heat stress decreases metabolic flexibility in skeletal muscle of growing pigs. Am. J. Physiol. Regul. Integr. Comp. Physiol..

[39] Akbarian A., Michiels J., Degroote J., Majdeddin M., Golian A., De Smet S. (2016). Association between heat stress and oxidative stress in poultry; mitochondrial dysfunction and dietary interventions with phytochemicals. J. Anim. Sci. Biotechnol..

[40] Biedasek K., Andres J., Mai K., Adams S., Spuler S., Fielitz J., Spranger J. (2011). Skeletal muscle 11beta-HSD1 controls glucocorticoid-induced proteolysis and expression of E3 ubiquitin ligases atrogin-1 and MuRF-1. PLoS ONE.

[41] Ma B., Zhang L., Li J., Xing T., Jiang Y., Gao F. (2021). Heat stress alters muscle protein and amino acid metabolism and accelerates liver gluconeogenesis for energy supply in broilers. Poult. Sci..

[42] Pedroso F.E., Spalding P.B., Cheung M.C., Yang R., Gutierrez J.C., Bonetto A., Zhan R., Chan H.L., Namias N., Koniaris L. (2012). Inflammation, organomegaly, and muscle wasting despite hyperphagia in a mouse model of burn cachexia. J. Cachexia Sarcopenia Muscle.

[43] Niu J.L., Zhang J., Wei L.Q., Zhang W.J., Nie C.X. (2019). Effect of fermented cottonseed meal on the lipid-related indices and serum metabolic profiles in broiler chickens. Animals.

[44] Sahebi-Ala F., Hassanabadi A., Golian A. (2021). Effect of replacement different methionine levels and sources with betaine on blood metabolites, breast muscle morphology and immune response in heat-stressed broiler chickens. It. J. Anim. Sci..

[45] Azad M.A., Kikusato M., Maekawa T., Shirakawa H., Toyomizu M. (2010). Metabolic characteristics and oxidative damage to skeletal muscle in broiler chickens exposed to chronic heat stress. Comp. Biochem. Physiol. A Mol. Integr. Physiol..

[46] Xue B., Song J., Liu L., Luo J., Tian G., Yang Y. (2017). Effect of epigallocatechin gallate on growth performance and antioxidant capacity in heat-stressed broilers. Arch. Anim. Nutr..

[47] Zeng T., Li J.J., Wang D.Q., Li G.Q., Wang G.L., Lu L.Z. (2014). Effects of heat stress on antioxidant defense system, inflammatory injury, and heat shock proteins of Muscovy and Pekin ducks: Evidence for differential thermal sensitivities. Cell Stress Chaperones.

